# Understanding Wheat Starch Metabolism in Properties, Environmental Stress Condition, and Molecular Approaches for Value-Added Utilization

**DOI:** 10.3390/plants10112282

**Published:** 2021-10-25

**Authors:** Kyung-Hee Kim, Jae-Yoon Kim

**Affiliations:** 1 Department of Life Science, Dongguk University-Seoul, Seoul 04620, Korea; redanan@dongguk.edu; 2 Department of Plant Resources, College of Industrial Science, Kongju National University, Yesan 32439, Korea

**Keywords:** wheat, starch metabolism, environmental stress response, molecular marker, transformation

## Abstract

Wheat starch is one of the most important components in wheat grain and is extensively used as the main source in bread, noodles, and cookies. The wheat endosperm is composed of about 70% starch, so differences in the quality and quantity of starch affect the flour processing characteristics. Investigations on starch composition, structure, morphology, molecular markers, and transformations are providing new and efficient techniques that can improve the quality of bread wheat. Additionally, wheat starch composition and quality are varied due to genetics and environmental factors. Starch is more sensitive to heat and drought stress compared to storage proteins. These stresses also have a great influence on the grain filling period and anthesis, and, consequently, a negative effect on starch synthesis. Sucrose metabolizing and starch synthesis enzymes are suppressed under heat and drought stress during the grain filling period. Therefore, it is important to illustrate starch and sucrose mechanisms during plant responses in the grain filling period. In recent years, most of these quality traits have been investigated through genetic modification studies. This is an attractive approach to improve functional properties in wheat starch. The new information collected from hybrid and transgenic plants is expected to help develop novel starch for understanding wheat starch biosynthesis and commercial use. Wheat transformation research using plant genetic engineering technology is the main purpose of continuously controlling and analyzing the properties of wheat starch. The aim of this paper is to review the structure, biosynthesis mechanism, quality, and response to heat and drought stress of wheat starch. Additionally, molecular markers and transformation studies are reviewed to elucidate starch quality in wheat.

## 1. Introduction

**Figure 1:**
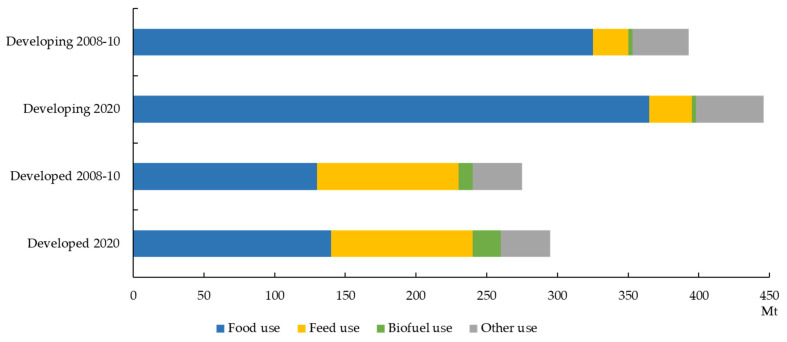
Wheat consumption in developed and developing countries. “Other use” refers to industrial uses of wheat. Source: OECD and FAO secretariats.

Wheat is a major cereal crop that provides the world’s population with calories and protein. Total wheat utilization is expected to reach nearly 746 million tons by 2020, and about 68% of total wheat use is projected to be consumed primarily as food by 2020 (Figure 1) [1]. Wheat is mostly used as food, seeds, feed, and fuel. Wheat grain is composed of 13% water, ~70% carbohydrates, 7~15% proteins, and 1.5~2% lipids [2]. In particular, wheat grains contain an important protein called gluten, which is needed in the basic structure to form a dough system for bread, cakes, cookies, cereals, pasta, and noodles. Among the different wheat species, *Triticum aestivum* is used to make bread and noodles, and *T. durum* for spaghetti and macaroni. *T. monococcum*, *T. dicoccum,* and *T. spelta*, customarily referred as einkorn, emmer, and spelt, respectively, are some of the ancient species used in grain berries, farro, and salads [3].

Wheat starch is an important by-product of gluten production [2]. Wheat endosperm is composed of about 70% starch. The difference in the quality and quantity of starch affects the flour processing characteristics. Wheat starch is obtained by removing protein from flour and is comparable to corn starch or flour in its processed state. It is now an essential part of the food industry and is mainly used as a food additive, which is an active ingredient in many food products. Currently, starch is also a major industrial material and is generally used in textiles, paper, and chemical and pharmaceutical industries as a thickener, stabilizer, gelling agent, and adhesive [3]. This diversification of wheat grain uses has given wheat breeders the idea of developing wheat varieties with grain characteristics suitable for new applications. In particular, the two types of granules (amylose and amylopectin) have different physicochemical properties [4]. Correcting the properties of wheat starch by genetic engineering alters the expression level of starch biosynthetic enzymes. This review explains the characteristics of wheat starch, the development of starch-related markers, and the research on wheat starch using biotechnology.

## 2. Characterization of Wheat Starch

**Figure 2:**
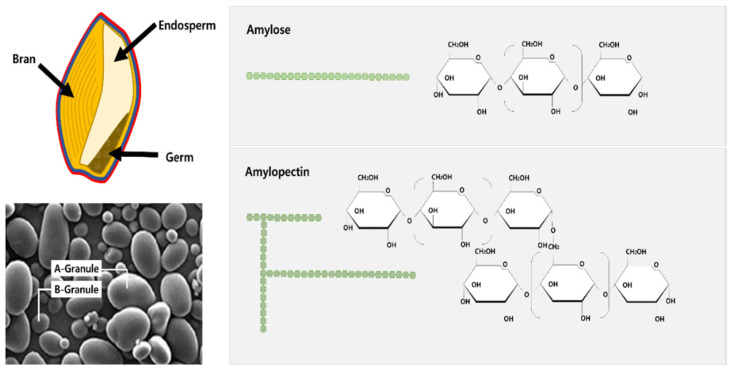
The main carbohydrates in wheat grain and flour: starch (amylose and amylopectin). Source: This figure was cited and edited in Shewry et al. [5].

Wheat starch is a major storage carbohydrate and contains about 60~75% grain and 70~80% flour [3]. Starch granules located in starchy endosperm cells are composed of two polymers called amylose and amylopectin (Figure 2) [5]. Starch consists of two granules, a large A-granule (5~40 µm) and a small B-granule (<10 µm). Amylose is a linear α-1, 4 glucan, comprising 25~30% of wheat grain starch [6]. Amylopectin is a highly branched larger glucan comprising 70~75% of wheat grain starch. Moreover, starch also contains relatively small amounts of minerals, which are not functionally significant, except for phosphorus [7]. Phosphorus is found mostly in three main forms, i.e., phosphate monoester, phosphate, and inorganic phosphate. Phosphate monoesters are bonded to a specific region within the amylopectin molecule [8,9]. 

Starch plays a significant role in the texture of many kinds of food and serves as a major source of energy for humans [10]. Native starch does not have a functional character for food treatment requirements such as thickening and stabilization. Therefore, starch used in the food industry is modified during processing and storage to overcome undesirable changes in the product textures due to the decomposition of starch. 

The waxy starch used in the food industry is typically chemically modified [11]. Cross-linked waxy starch typically exhibits shorter textures, higher paste stability, cooking shear, temperature and resistance to lower pH than native starch [12]. Modified starch made from waxy wheat has a lower gelatinization temperature and paste clarity than the modified starch made from corn starch. However, the freeze–thaw stability of modified wheat starch is generally better than modified waxy corn starch [13]. 

### 2.1. Starch Structure

Starch granules are composed of two types of α-glucan, amylose and amylopectin, and make up about 98-99% of the dry weight [7]. The proportion of both polysaccharides varies depending on the plant origin of the starch. "Waxy" starch contains less than 15%, ("normal" 20-35%), and "high" (amylo-) amylose starch contains more than 40%. Amylose and amylopectin (Figure 2) differ in structure and properties and have been discussed and reviewed by many authors [14,15,16,17]. Amylose is a relatively long linear α-glucan containing about 99% of (1→4)-α- and (1→6)-α- linkages and differs in size and structure according to plant origin. The molecular weight of amylose is approximately 1 × 10^5^~1 × 10^6^ [17]. Amylopectin (Figure 2) has a molecular weight of 1 × 10^7^–1 × 10^9^ [17,18] and is much larger than amylose. Amylopectin is a heavily branched structure consisting of about 95% (1→4)-α- and 5% (1→6)-α-linkages. The starch is a main component in wheat grain, which accounts for 60–70% of its dry weight [19], followed by protein, which defines the grain quality [20]. The wheat kernel starch in the endosperm has three types (A-, B-, and C-type) of starch granules, each distinguished by its properties [20]. Each type has a unique physiochemical property that determines the quality of starch. The dynamics of starch granule size distribution, the activity of starch synthase, and the expression of genes encoding starch synthase were studied in superior and inferior grains during grain filling. The superior grains showed higher grain weight, starch, amylose, and amylopectin contents compared to inferior grains. The genotype X environmental interactions affect the polymers and alter grain starch and protein formation [21]. Recently, the effects of climate change on grain quality and food safety have been considered. The content and quality of wheat protein is also affected by plant nutrition and crop management. Additionally, under elevated temperatures between anthesis to grain maturity, the grain yield is reduced because of the reduced time to capture resources [22].

### 2.2. Starch Biosynthesis Mechanism

**Figure 3:**
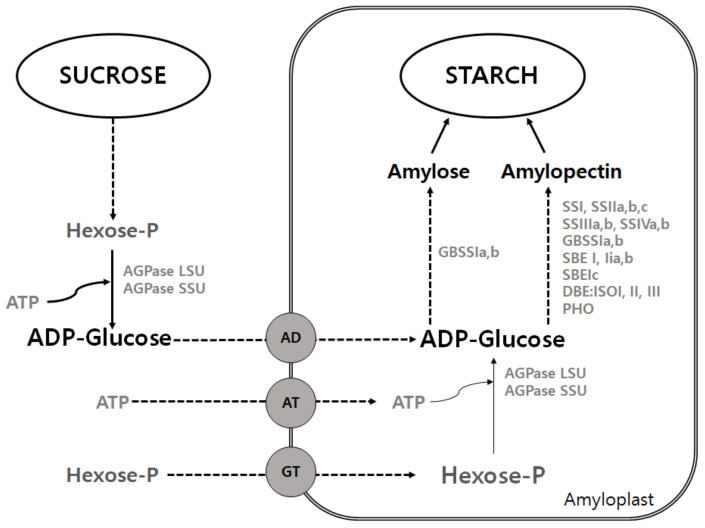
Outlines of starch biosynthesis pathway. ADPglucose pyrophosphorylase (AGPase); AGPase large subunit (AGP-L); AGPase small subunit (AGP-S); ATP/ADPglucose transporter (AD); plastidial ATP transporter (AT); glucose-6-phosphate transporter (GT); starch synthase (SS); granule-bound starch synthase (GBSS); starch branching enzyme (SBE); starch debranching enzyme (DBE); starch phosphorylase (PHO). Source: This is an overview cited in Thitisaksakul et al. [26].

Starch is the main storage compound in plants, present in both production and storage organs. As starch biosynthesis is a complex process [7,17], higher plants use prokaryote-like starch biosynthetic pathways for the formation of adenosine 5’-diphosphate glucose (ADP-glucose) [23], a soluble precursor and substrate for starch synthase [24]. ADP-glucose initiates the starch biosynthesis by the action of the enzyme ADP-glucose pyrophosphorylase (AGPase, E.C. 2.7.7.27), which catalyzes the reaction of glucose-1-phosphate with ATP in the plant cells [25]. The AGPase reaction is the first step carried out in the biosynthesis of transient starch in chloroplasts and chromoplasts, and subsequently imported into amyloplasts, following different mechanisms of post-translational regulation by related genes. The biosynthetic pathway for starch is summarized in Figure 3 [26]. 

Sucrose produced by photosynthesis moves to the amyloplast and is metabolized to hexose phosphate. These hexose phosphates act as a substrate for starch, protein, and oil biosynthesis. When the endosperm develops, most of the hexose phosphate is used for starch biosynthesis. In order to induce such an energy-intensive reaction, phosphorylation and ATP production are required. 

**Figure 4:**
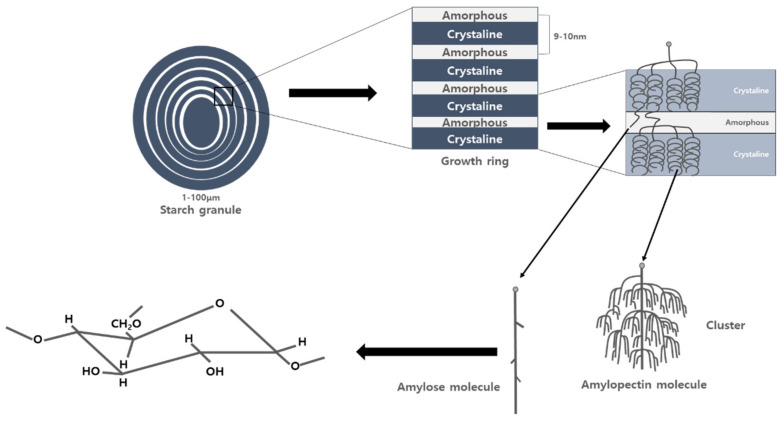
Schematic diagram of the various structural levels of starch granules and the relationship between amylose and amylopectin. Source: This schematic is cited from Buléon et al. [17].

Starch synthase enzymes separate glucose residues from ADP-glucose and bind them to the ends of amylose and amylopectin to elongate polymer polysaccharide chains. In the polysaccharide chain constituting amylose, the OH groups of carbon 1 and carbon 4 of glucose inside the chain are continuously connected. Amylopectin shows a regular branch shape by connecting the OH groups of carbon 1 and 6 in addition to the polysaccharide chain of amylose. The formation of these branches involves the starch branching enzyme (SBE) [26]. These two polymeric compounds form semi-crystalline starch granules, where the exact proportion, size, and shape of the starch granules vary according to plant species and organs [16]. A schematic diagram of the granular structure is shown in Figure 4.

When the endosperm of wheat, corn, barley, and rice is developed, the cytosolic isoform of AGPase accounts for 65 to 95% of the total AGPase activity [25]. In higher plants, AGPase is a heterotetramer, consisting of two large (AGP-L) subunits and two small (AGP-S) catalytic subunits encoded by two or more different genes [27]. Plants have multiple genes that encode AGP-L or AGP-S subunits, which are differentially expressed in different plant organs. The multiple genes encoding AGP-L subunits show strong specificity in expression as they are limited to leaves, roots, and endosperm of barley [28], wheat [29], and rice [30], or derived from certain conditions, such as increased sucrose or glucose levels in potatoes [31,32].

### 2.3. Resistant Starch Wheat

We consume grains, pasta, and potatoes in our daily lives, and most of these carbohydrates are starch. Starch quality is largely determined by the ratio of amylose to amylopectin [22]. Normal starch is rapidly digested and absorbed as glucose. During this process of digestion, our body produces a hyperglycemic response. To alleviate this, insulin is secreted, and the process of becoming hypoglycemic again is repeated. If this process is repeated, our body is easily exposed to various diseases such as obesity and diabetes. This is why carbohydrates are considered a public enemy. Resistant starch, a type of starch, is a carbohydrate with an inverted attraction. Resistant starch is starch that is not easily broken down by digestive enzymes in the body [33]. Amylase cannot break it down into glucose, so it is not absorbed by the body. Instead, it is broken down by bacteria in the large intestine. A type of starch called resistant starch (RS) is not easily decomposed by digestive enzymes in the body. Starch with an increased amylose ratio is of great interest because it contributes to RS in food and has a beneficial effect on human health. Recently, high amylose starch showed a positive effect on health in a study related to obesity in humans [34]. RS can generally be divided into five types [5,35]: 1) types such as seeds, legumes, and whole grains that are difficult to digest; 2) types that contain a lot of resistant starch when raw, but which disappears when ripening; 3) low resistant starch content when warm after cooking, but low in resistant starch content when cooled (higher varieties); 4) chemically manufactured starch varieties; 5) a type that combines with the type of fat to change its structure and improve digestion. Foods rich in resistant starch include oats (oatmeal flakes), cold rice, cooked legumes, cooked potatoes, and unripe bananas. Unripe bananas, for example, contain about 20% resistant starch. The resistant starch in bananas helps in weight loss because it stimulates glucagon, which promotes fat breakdown without raising blood sugar.

In 1982, during in vitro analysis of non-starch polysaccharides, Englyst and workers discovered that some starch remained after enzyme hydrolysis [36,37,38]. A follow-up study of healthy ileostomy confirmed that similar starch resists digestion in the stomach and small intestine. Further analysis has shown that such starch can be fermented in the large intestine. This type of starch is named resistant starch (RS) [39]. RS can reach the colon and serve as a substrate for microbial fermentation, and its final products are hydrogen, carbon dioxide, methane, and short chain fatty acids [40]. According to Wong et al. [35], resistant starch acts similarly to dietary fiber, nourishing intestinal bacteria and increasing the production of short chain fatty acids such as butyrate. Brouns et al. [41] also found that resistant starch had the effect of keeping mucosal cells in the colon healthy and preventing cancer cell division. Based on the causes of enzyme resistance, RS is classified into five different types [42,43]. Table 1 summarizes the different types of RS, their classification criteria, and food sources.

RS1 is starch that is physically inaccessible to digestion, such as that in whole grains or tubers. RS2 is a native starch granule that is protected from digestion by the conformation or structure of the granule and is found in raw potatoes and green bananas. RS3 is retrograded starch formed when starchy foods (e.g., potatoes, pasta) are cooked and then cooled. Cooling allows the amylose and linear parts of amylopectin to form crystalline structure that reduces digestibility. RS4 is chemically modified starch formed by crosslinking, etherization, or esterification and it is found in foods containing modified starches such as some bread and cakes. RS5 is a starch wherein the amylose component forms complexes with lipids (amylose–lipid complex). The amylose–lipid complex is generally found in native starch granules and processed starch. This complex also entangles amylopectin molecules, restricting the swelling of starch granules and enzyme hydrolysis [47,48]. The formation of amylose–lipid complexes is an immediate reaction, and RS5 is considered thermally stable because the complex can regenerate after cooking [49]. The presence of the amylose–lipid complex in starch granules increases their enzyme resistance by restricting granule swelling during cooking [47]. 

## 3. Wheat Starch under Heat/Drought Stress

### 3.1. Grain Filling Stage under Heat/Drought Stress

Wheat development stages include “germination, emergence, tillering, floral initiation, terminal spikelet, stem elongation, spike emergence, anthesis and maturity” (http://www.fao.org). Starch synthesis was observed, that this mechanism was strongly relevant during the stages of anthesis and maturity [50]. The wheat starch production losses are caused more by abiotic stresses such as drought and high temperature than by biotic insults or other abiotic stresses [51]. Thus, understanding the effects of these stresses becomes indispensable for wheat starch improvement programs that have depended mainly on environmental factors such as heat and drought stresses.

Wheat plants are frequently subjected to varying degrees of heat and drought stress during their growth stage [52]. Heat stress is assessed by the degree and rate of temperature rise, as well as the amount of time spent exposed to the elevated temperature [53]. Globally, the rise in daily minimum temperatures was more than twice that of daily maximum temperatures between 1950 and 1995 [54]. Greater temperature variability and an increase in the frequency of warm days will also have an impact on future climates [55]. Wheat yield losses will increase by up to 30% by 2050 as a result of climate change and a 2-3°C rise in global temperature [56]. Optimal temperature for wheat anthesis and grain filling period is between 20 and 25°C. Wheat grain filling rate is reduced when exposed to temperatures above 30°C during the anthesis and grain filling stages, resulting in lower yield and quality [57,58]. As a result, heat stress is a significant challenge to wheat production and optimal yields [59]. Drought stress decreases cell elongation and growth by causing water loss, turgor loss, and stomatal closure [60,61]. It also causes early senescence and reduces the duration of grain filling stage since photosynthesis trigger and metabolism is disrupted, resulting in death of cell [62]. Therefore, it is important to elucidate wheat tolerance mechanisms in response to drought and high-temperature stress during the grain filling period. During the reproductive phase of wheat growth, drought and high-temperature stress has emerged as a serious problem for starch synthesis. In the case of Korea and other countries, anthesis and maturity periods were mostly from April to June. In Miryang, one of Korea’s largest wheat cultivation areas, rain precipitation has continued to decrease, while the temperature continues to increase during the anthesis and maturity period for the past three years (rainfall : 2018 (93.5 mm), 2019 (50.3mm), 2020 (48.6mm) / Temperature : 2018 (28.5 °C), 2019 (29.1 °C), 2020 (30.2 °C)) (https://data.kma.go.kr/). To overcome these problems, early ripening wheat cultivars through crossbreeding (Jokyung (accession no. 102005000184), Jopum (102000200523), Joeun (102001000044) etc.) were released from the 1990s to 2010s in Korea. Additionally, transcriptome analysis under heat stress was performed on Korean wheat cultivars during the ripening period [63].

### 3.2. Starch & Drought and Heat Stress during Anthesis and Grain Filling Stage

Starch is more sensitive than storage protein to heat stress [64]. Although wide genetic variability was observed among the wheat species for heat tolerance in grain starch content [65], changes in amylose and amylopectin deposition, as well as changes in starch granule formation are of specific importance [26]. During the wheat grain filling period, high temperatures reduced the starch content and modified the size distribution of starch granules in grains [6]. This also changed the chain length of amylopectin in endosperm starches [66] and caused poor starch granule structure [67]. Starch synthesis is highly susceptible to high-temperature stress due to the susceptibility of the soluble starch synthase in developing wheat kernels [68,69]. During the grain filling period, short periods of very high temperature (35–40°C) could have a negative impact on grain quality [70]. However, the high-temperature acclimation effectively improved the carbohydrate remobilization from stems to grains during anthesis. This resulted in less modified starch content and starch granule size distribution in wheat grains [20]. Since pollen maturation necessitates the use of starch as an energy reserve, starch accumulated in stem tissue is used as a temporary soak during the reproductive process of plants [71]. Pollen production is interrupted, and pollen mortality is increased as a result of a high-temperature-induced impediment in starch mobilization within the anther [72]. Drought stress can cause grains to lose their total starch and amylopectin content during the flowering stage [73]. However, it affects starch size distribution and branch chain length during anthesis [74]. Under both stress conditions, drought reduced the size of small starch granules, while heat stress reduced the size of large granules. Thus, the changes in morphology and size distribution of starch granules resulted in a decrease in starch content and total grain yield [75]. Despite the significant deleterious effect of high-temperature and drought stress on wheat production, the plant’s starch response mechanism during the grain filling period could not be clearly elucidated.

### 3.3. Starch & High Night Temperature during Anthesis and Grain Filling Stage

High temperatures of 20 to 23°C at night reduced the grain-filling period by 3 to 7 days [76]. There has recently been a critical decrease in the rate of grain filling in wheat cultivars when the day/night temperature is 32/22°C compared to 25/15°C [77]. High temperatures of 31/20°C during the day and night can cause changes in the aleurone layer and endosperm structures [78]. Higher night temperature reduced the transcript levels of the adenosine diphosphate glucose pyrophosphorylase small subunit but increased the starch-degrading enzymes isoamylase III, alpha, and beta-amylase by a factor of two in developing grains [79]. Likewise, the increase in night temperature shortens the grain-filling period and reduces the grain structure more so than that of day temperature. To improve wheat yield and quality under heat stress, a thorough study of grain weight stability in terms of starch components between day and night temperatures is needed.

### 3.4. Sucrose and Starch Biosynthetic Pathway Carbohydrate Metabolism under Stress

Heat stress decreases cereal starch content while increasing protein content during grain filling [80]. It did not affect the swelling power or starch solubility of wheat starches, but it did significantly reduce the swelling ability of wheat flours and enzymatic digestibility of wheat starches [81]. Sucrose is processed by invertases, sucrose synthases, and sucrose phosphate synthase after it enters the grain [82]. The activity of these enzymes appears to be an effective target for control in wheat under heat stress to improve grain filling processes and yield [83]. Multisite protein phosphorylation modulates sucrose phosphate synthase in response to temperature [84]. In developing pollen grains, heat stress inhibits sucrose synthase, as well as many cell walls and vacuolar invertase. As a result, the sucrose and starch turnover is impaired, and soluble carbohydrates accumulate at lower levels [85]. Hence, there is the necessity to analyze the sucrose and starch biosynthetic pathway mechanism under heat stress.

### 3.5. Carbohydrate Metabolism under Stress

During heat stress, carbohydrate availability is a significant physiological feature linked to heat stress resistance [86]. Survival strategies of plants subjected to environmental influences such as high temperature depend on efficient carbohydrate metabolism as a source of energy and carbon skeletons [87]. Due to changes in photosynthetic carbon metabolism, heat stress prevents plant development, disrupts mineral–nutrient relationships, and impairs metabolism [88]. Invertase is required for the hydrolysis of sucrose into glucose and fructose. A central enzyme in sucrose metabolism, Cell Wall Invertase (CWIN), catalyzes the irreversible breakdown of sucrose into glucose and fructose, and downregulates the genes involved in carbohydrate metabolism under heat stress [89]. Drought stress inhibits plant growth, disrupts mineral–nutrient relationships, and impairs metabolism due to changes in photosynthetic carbon metabolism [90]. It is well known that stress can alter the activity of an enzyme, and the changes to sucrose-metabolizing enzyme activities also modify the sucrose metabolism in leaves. However, no consistent conclusion on the impact of stress on sucrose metabolism has been drawn, and various studies have reached different conclusions.

### 3.6. Regulation of Starch Metabolism under Stresses

Starch metabolism enzymes consist of sucrose phosphate synthase (SPS), sucrose synthase (SuSy), ADP-glucose pyrophosphorylase (AGPase), glucokinase, soluble starch synthase (SSS), and starch branching enzyme (SBE) [91]. Heat stress during grain filling decreased these activities of enzymes, which restricted the accumulation of starch [91]. The functions of these main enzymes, as well as their genes associated with the conversion of sucrose to starch, were decreased, which was the major cause of starch content reductions [92]. AGPase is one of the enzymes that is presumed to be the primary site of starch deposition regulation in storage tissue [93]. Sucrose-6-phosphate synthase activity was measured in mature leaves, and sucrose synthase, AGPase, and UDP-glucose pyrophosphorylase activities were measured in the growing tubers of plants. Tuber sucrose synthase and ADP-glucose pyrophosphorylase activity were decreased but at a slower rate than leaf sucrose-6phosphate synthase activity [94]. Sucrose synthase and adenosine guanine pyrophosphorylase activity is high in growing tubers but decreases as tubers mature [95]. Heat stress increased the accumulation of foliar sucrose and decreased starch accumulation. Drought conditions influence the activities of starch biosynthesis enzymes such as GBSS, SS, and ADP-glucose pyrophosphorylase (AGP) [26]. Hexokinase catalyzes committed steps in glucose metabolism by forming hexose phosphate [96]. In both hexokinase-dependent and independent pathways, glucose serves as a signal molecule in addition to its structural function [97]. Drought stress increased the expression of two hexokinase transcripts [98]. Heat and drought stress suppressed the starch deposition by lowering the activity of all enzymes involved in starch synthesis except hexokinase.

### 3.7. Starch Synthetic Metabolism under Stresses

Heat stress reduced the activities of SPS and SuSy, resulting in lower sucrose levels during the grain filling period [99] and increased the activities of SuSy and SBE during the early stages of grain production but decreased subsequently [100]. It reduced the activities of enzymes involved in starch synthesis (AGPase, SSS, and SBE) and suppressed the grain weight and starch deposition during the grain filling period [101]. It also has a negative influence on SSS activity and starch granule synthesis [102]. SSS is highly sensitive to high temperatures [103], with relatively tolerant cultivars having higher catalytic efficiency of SSS at elevated temperatures and higher heat shock protein content (HSP 100). The relation between SSS activity at higher temperatures and HSP 100 levels in wheat grains may be due to SSS denaturation defense mechanism [104]. Limit dextrinase (LD) is the only endogenous hydrolase that can cleave α-1−6 linkages amylopectin and β-limit dextrin [105]. Lower LD activity results in lower fermentable sugar production and a higher level of dextrin [106]. The LD activity decreased sharply during thermo treatment [107]. As such, heat stress causes a decrease in several enzymes involved in the starch synthesis mechanism. Drought treatments reduced LD activity in all genotypes, but the degree of the reduction differed by genotype and treated time [108]. The production of endosperm starch granules and the physicochemical properties of starches may be affected by drought, affecting the consistency of final wheat products [109]. Both heat and drought stresses, which have a great influence on the grain filling period and anthesis, also have a negative effect on starch synthesis in combination.

### 3.8. Starch and Other Stresses during Anthesis and Grain Filling Stage

In the case of India and China, waterlogging limited wheat production [110]. Total rainfall was up to 500-800mm from March to May, coinciding with anthesis and maturity, which made the starch of wheat grain [111]. After anthesis, waterlogging caused poor production [112]. Waterlogging reduces grain number and grain weight depending on exposure waterlogging time lapse [113]. GBSS activities were declined as was ADP-Gppase under water stress [114]. Waterlogging affected several starch properties via downregulating the expression of soluble starch synthase, amylopectin content, and number of starch granules [115]. In another piece of research, waterlogging depressed ADP glucose pytophosphorylase and the amylopectin/amylose ratio [116]. As such, waterlogging also caused damage to the starch mechanism during the early anthesis period. 

Pre-harvest sprouting (PHS) is the premature germination of grain before harvest in wheat. PHS occurs in several wheat-growing regions [117]. When germination begins, causing starch and protein degradative enzymes to be produced, which break down endosperm starch and protein to germination [118]. Alpha amylase is a starch-degrading enzyme that is generated during the PHS process [119]. Increased endoprotease such as amylase, protease, and lipase activity in sprouted wheat causes protein or starch degradation, resulting in decreased wheat quality [120]. There are several strategies used to reveal PHS such as QTL [121] and MALDI TOF [122]. Because PHS induced several enzymatic reactions, genetic research was also needed.

One of the environmental factors that limits crop development and agricultural output is salt stress. High salinity has been shown to have an impact on carbohydrate metabolism. Salt stress induced GBSS expression that was highly controlled at the transcriptional level [123]. Salt stress is regulated by ADP-glucose pyrophosphorylase (AGP), starch synthase (SS), and starch branching enzyme (SBE) [124]. Synthesized triticale starch showed a decreased population of small granules and an increased ratio of A-type to B-type granules under salinity stress [125]. Salinity stress was observed to increase starch synthesis to regulate several enzymes; however, little is known about the molecular mechanism by which NaCl regulates starch accumulation.

## 4. Molecular Marker Development and Application for Wheat Starch

In the past, breeding research has relied on measuring only the characteristics of interest to select the superior lines. For example, it is easy to choose simple morphological properties such as plant height to select large amounts of offspring. Of course, the size and yield of each grain can also be considered. However, most of these characteristics require laboratory analysis or bioassay. Many characteristics are difficult to measure (e.g., grain dormancy and late maturity), so the resources available to breeders impose significant constraints on the speed and scale of their choice. In such cases, the use of markers is of great value to wheat breeders who indirectly represent the characteristics of interest and are relatively easy to score [126]. Markers can be linked (i.e., likely inherited with genetic proximity of markers and gene-dependent properties of interest) or diagnosed if they are directly related to genes. These diagnostic markers do not require independent verification for each parent line used in breeding programs and have an important advantage of having an absolute association with the selected characteristics. 

In order to develop an efficient breeding program in common wheat, four techniques (SDS-PAGE, 2-DE, MALDI-TOF-MS, and PCR) were compared to evaluate the suitability [127]. Of these, PCR-based markers showed the easiest, most accurate, and rational technique, recommending the identification of Glu-A3 and Glu-B3 alleles in breeding programs. Seventeen allele-specific markers have been reported for the Glu-A3 and Glu-B3 loci (Table 2), and, in fact, multiple PCR protocols have been developed to reduce screening costs in breeding programs [128].

Additionally, the application of functional markers for the identification of LMW-GS in wheat germplasm of various types has been reported [143]. Functional markers are developed from a functional polymorphism in the gene coding sequence, which can be a single nucleotide polymorphism (SNP) or InDels [142]. Map-based cloning and micro-mapping are the most effective strategies to isolate functional genes from plants [144].

Molecular marker technology has provided a new and efficient tool to improve the quality of bread wheat. To improve and support bread-making quality, high-throughput Kompetitive Allele-Specific PCR (KASP) analysis was performed and verified for key genes including the *wbm* gene on the 7AL chromosome and the overexpressed glutenin Bx7OE (*Glu-B1al*) gene [141]. These high-throughput marker resources have provided and made available the opportunity to improve bread-manufacturing quality in wheat breeding. As a PCR-based marker for each allele of waxy wheat, genes such as Wx-A1, Wx-B1, and Wx-D1 can identify wild-type and null waxy alleles at the waxy locus [139,145,146]. These PCR marker sets were used to identify and characterize waxy mutations occurring in the Wx-A1, Wx-B1, and Wx-D1 genes of 168 wheat lines [147].

An important factor in determining the amylose content of grain starch is the 59 kDa granule bound starch synthase (GBSS) protein [148,149]. In wheat starch, amylose levels are affected by the activity of GBSS1 in the process of endosperm development [150]. Low amylose content in wheat has the effect of increasing starch viscosity and flour swelling volume (FSV) [151,152], and this property is preferred for white salt (udon style) noodle production [153,154]. In durum wheat, the Wx-B1 null mutation resulted in decreased amylose content with increased starch dough viscosity and FSV [155]. In addition, pasta derived from the Wx-B1 null line had lower cooking losses. Furthermore, cooking losses have shown a correlation with amylose content, peak starch viscosity, swelling power of semolina, and adhesiveness of cooked pasta [155]. 

 Two types of GBSS genes, GBSSI and GBSSII, are present in wheat (*T. aestivum* L.), barley (*Hordeum vulgare* L.), corn (*Zea mays* L.), and rice (*Oryza sativa* L.) [156]. The GBSSI gene responsible for amylose synthesis in endosperm tissue is located at the waxy locus, and the GBSSI gene product is known as the Waxy (Wx) protein [157]. Waxy (GBSSI triple null) particles can be identified by a simple potassium iodide staining [158]. Each GBSS protein can be detected by 2D-electrophoresis [139] and SDS-PAGE under optimal conditions [159]. In wheat, three GBSSI genes located on chromosomes 7A (Wx-A1), 4A (Wx-B1), and 7D (Wx-D1) encode GBSSI. In the absence of the GBSS enzyme in the grain endosperm, this tissue consists almost entirely of amylopectin [158]. Meanwhile, in order to identify wheat with the desired texture for udon noodles, a specific PCR analysis method was developed to identify molecular markers linked to the GBSS 4A locus. [160]. These PCR markers can be tested easily and accurately because they use the leaves of young seedlings or mature seeds compared to conventional methods used to screen the quality of udon noodle starch. In addition, this PCR marker analysis is advantageous to identify a breeding line that is heterogeneous for the 4A allele. 

With the development of Next-Generation Sequencing (NGS), NGS-based genotyping techniques were applied to the development of molecular markers for grain starch or quality [161]. A genome-wide association study (GWAS) is considered an attractive approach for assessing grain quality. Starch contents and starch-related parameters in rice were studies using GWAS analysis [162,163]. Recently, GWAS was performed to identify genetic factors of wheat grain quality including grain protein content, grain starch content, and grain hardness [164]. This kind of studies, especially GWAS analysis for wheat quality, could become a growing trend in digital big data-based precision breeding.

## 5. Genetic Modification of Starch Composition in Wheat

Grain is the part harvested from wheat, and its nutrition and properties are determined by its biochemical composition. In wheat seeds, starch accounts for 55 to 75% of the total dry grain weight and contains 10 to 15% of the storage protein. In addition, starch and protein have a significant impact on the quality of products made from flour. Optimal starch and protein, and the right levels of essentials (iron, zinc, calcium, phosphorus, and antioxidants) are indispensable elements in healthy wheat products. Most of these quality traits in recent years have been developed for research using genetic modification interventions.

The starch portion, which comprises about 70% of the total dry matter of wheat grains [165], can have a significant impact on products made from wheat kernels. For example, the quality of noodles manufactured from flour depends primarily on the characteristics of starch [166]. The physicochemical properties and end uses of wheat starch are related to starch structure and the distribution of two major glucan macromolecules, amylose and amylopectin [167]. A clear strategy for modifying the properties of wheat starch by genetic engineering involves changes in the level of starch biosynthetic expression. 

Wheat starch granules accumulate at least three types of starch synthase (SS) with molecular masses of about “60, 77, 100 to 115 kDa” [159]. Most SS synthesis appears to be in the soluble portion of the endosperm [168]. Several genetic studies of mutations lacking ~60 kDa granule-bound SS (GBSS1; waxy protein) in cereals strongly suggest their role in amylose synthesis [158,169].

Temporary starch produced in photosynthetic tissue may not be fully applicable to starch build-up in sink parts, but the same enzymatic function is also believed to be involved in starch biosynthesis in both localities [93]. Genes encoding starch biosynthetic enzymes can affect the fine structure of starch, with differences in spatial and temporal regulation, substrate specificity, concentration, and movement. To further understand the starch biosynthesis processes in wheat, molecular strategies to change the level of expression of starch biosynthesis genes have been adopted in combination with plant breeding techniques [167]. The new information collected from hybrid and transgenic plants is expected to help develop novel starch for understanding wheat starch biosynthesis and commercial use.

Traditional breeding techniques or genetic modification can be used to produce novel starch with modified properties [170]. Using genetic modification techniques, high amylose starch (starch with up to 70% amylose content) and wax starch (99–100% amylopectin content) were produced [171]. It also produced starch that transformed the amylopectin structure by adjusting the phosphate content and granule size. Currently, research on wheat transformation using plant genetic engineering technology is reported to constantly control and analyze the characteristics of wheat starch (Table 3).

RNA interference (RNAi) is a powerful tool for functional gene analysis and engineering of novel phenotypes, which is a common regulatory mechanism for gene expression in eukaryotic cells. This technique directs gene silencing after transcription in a sequence-specific manner based on the expression of antisense or hairpin RNAi constructs, or other forms of short interfering RNA molecules. The application of RNAi contributed to the manipulation of wheat particle size [185,186] and quality [187,188]. The NAC gene that controls aging improves the grain protein, zinc, and iron content of wheat [186]. The ancestral wild wheat allele encodes the NAC transcription factor (NAM-B1) to accelerate aging, while modern wheat varieties have a non-functional NAM-B1 allele. Thus, reduction in RNA levels of multi-NAM homologues by RNAi delayed aging by more than 3 weeks and reduced wheat grain protein, zinc, and iron content by more than 30%. The RNA interference expression vector of TaCKX2.4 was constructed and transformed in bread wheat NB1, and the number of grains per spike was improved due to RNAi of the cytokinin oxidase 2 (*CKX2*) gene in the transgenic line [187]. That is, the expression level of *TaCKX2.4* was negatively correlated with the number of grains per spike, and the number of grains per spike was increased in wheat with decreased *TaCKX2.4* expression. RNA silencing of the waxy gene by the RNAi strategy confirmed a decrease in amylose levels in transgenic wheat seeds [188]. According to iodine staining and amylose content analysis in these transgenic seeds, the level of amylose in the endosperm was significantly reduced in transgenic seeds. In addition, RNAi was used to suppress the expression level of the 1Dx5 high-molecular-weight glutenin subunit, resulting in a transgenic wheat line [189]. The silence of the 1Dx5 expression significantly reduced the quality of flour processing based on Farinograph, Gluten, and Zeleny tests. Consequently, it was found that RNAi is useful for silencing the HMW-GS gene.

Silencing of the *SBEIIa* gene increased the amylose content in durum wheat [184]. The starch granules of these transgenic lines have a deformed, irregular, constricted shape, and are smaller than the unmodified control. In durum wheat, silencing of the *SBEIIa* gene causes changes in granule morphology and starch composition, resulting in high-amylose wheat. High-amylose durum wheat was produced through mutagenesis of starch synthase II (SSIIa or SGP-1) [182]. Therefore, high-amylose durum may be useful for making valuable pasta with increased elasticity and reduced glycemic index. An EMS-induced mutant population for amylose and resistant starch mutations of bread wheat (*T. aestivum*) was developed, and candidate genes responsible for the amylose mutation were identified [173].

Starch composition, structure, and properties were modified through editing of TaSBEIIa in both winter and spring wheat varieties using CRISPR/Cas9 [177]. TaSBEIIa determines the starch composition, structure, properties, and end-use quality across a variety of genetic backgrounds. It also improves RS content through multiple breeding and end-use applications in grain crop species, thus utilizing genome editing for health benefits. Novel NAC transcription factors, TaNAC019-A1 (TraesCS3A02G077900) and NAC019-A1, negatively regulate starch synthesis in wheat and rice (*Oryza sativa* L.) endosperm, and provide new insights into improving wheat yield (citation). *TaMTL* was edited using an optimized *Agrobacterium*-mediated CRISPR system to efficiently induce haploid plants in wheat [174]. Two endogenous genes, *TaWaxy* and *TaMTL*, were edited with high efficiency by the optimized SpCas9 system, and the highest efficiency (80.5%) was achieved when targeting *TaWaxy* using TaU3 and two sgRNAs.

After genetically modified organisms (GMOs), the era of ’gene-edited crops’ is coming. The United States, Canada, Israel, Japan, and Australia have already begun to approve the production of gene-edited crops. Targeted gene editing, especially CRISPR/Cas9, is a tool with significant potential for plant development and breeding [190,191]. Gene-edited crops are one kind of crop in which DNA is deleted or inserted to improve genetic traits within an organism other than a foreign gene by techniques such as gene scissors. This technique can be used to enhance the good nutrients of a crop or remove the bad nutrients. Gene editing is a transient step that enables editing of a target gene, requiring the introduction of foreign DNA (a zinc finger protein, TALEN, or a structure plus guide RNA for Cas9 and CRISPR/Cas9) or protein into the plant genome or plant cell [192]. Foreign DNA is isolated from the next generation and is not present in the final gene editing line and final product. To address these issues, several approaches must be combined, and, almost certainly, genes edited from different lines must be combined through crosses and selection within breeding programs. It is also suitable for determining the safety and quality of grains screened and produced during these breeding programs under stringent regulations. Additionally, the advent of genome editing has sparked enthusiasm, but, at the same time, it has sparked controversy and raised regulatory and governance concerns around the world. In gene-editing research, human embryos are subject to strict regulations due to ethical concerns, which poses challenges to research activities [193,194]. As agriculture faces major challenges to provide food and nutritional security, producing more food with sustainable production requires the development of crops that will significantly contribute to the achievement of several sustainable development goals [195]. In the case of plants, since ethical issues are somewhat insignificant, flexible regulation should be carried out. Moreover, transgene-free genome-edited plants can be easily generated by ribonucleoproteins (RNP) or Mendelian segregation [196,197]. Therefore, if policy and governance issues are addressed at national and international levels, plant genome editing can play a key role in developing useful crops, along with rapid scientific progress.

Kernel hardness, a quality characteristic of common wheat (*T. aestivum* L.), is primarily regulated by the *Pina* and *Pinb* genes. Mutation or deletion of *Pina* or *Pinb* increases kernel hardness, resulting in hard wheat kernels. Transformation of *Pinb-D1x* into soft wheat using bombardment technology produces a hard wheat kernel texture [179]. According to the data from the single kernel characterization system and scanning electron microscopy, the introduction of *Pinb-D1x* into the soft mill significantly increased the kernel hardness and changed the internal structure of the kernel. The low molecular weight glutenin subunit LMW-N13 improved the dough quality of transgenic wheat using Agrobacterium-mediated technology [175]. To analyze the contribution of LMW-N13 to dough quality, three transgenic wheat lines overexpressing LMW-N13 were generated. Compared to the non-transgenic (NT) line, the transgenic (TG) line showed excellent dough properties. These excellent dough properties resulted in higher glutenin macropolymer (GMP) and total protein content.

## 6. Conclusions

Wheat starch is an important by-product of gluten production, and wheat endosperm is composed of about 70% starch, so differences in the quality and quantity of starch affect the flour processing properties. Wheat starch, in particular, is the main storage carbohydrate and contains about 60 to 75% of grains and 70 to 80% of flour. In plants, starch is a major storage compound present in both production and storage organs, and starch is synthesized through a complex biosynthetic process. These starches are rapidly digested and absorbed as glucose. In the process of digestion, the human body responds to hyperglycemia. In order to relieve this, insulin is secreted and the process of becoming hyperglycemic is repeated. If we repeat this process, the body is more likely to be exposed to various diseases such as obesity and diabetes. Recently, many studies conducted on resistant starch (RS), a type of starch, proved that it is not easily decomposed by digestive enzymes in the body [198]. Resistant starch acts similar to dietary fiber, providing nutrients to intestinal bacteria. Cereals high in amylose content (AC) and resistant starch (RS) have potential health benefits [30,33]. Grains with higher amylose content (AC) are good sources of RS [199]. Grains high in RS are reported to help improve human health and reduce the risk of serious non-infectious diseases [172]. Currently, there is an increasing need for developing crops with high RS to address the rapidly growing nutritional challenges for public health [172,200,201]. Amylose and amylopectin are synthesized through two different pathways. Amylose synthesis requires active granular binding starch synthase (GBSS), whereas amylopectin is a complex pathway involving other isoforms including starch synthase (SS), starch branching enzyme (SBE), and starch debranching enzyme (SDBE) [202]. Starch synthesis can be directed to amylose production by overexpressing the appropriate GBSS (Waxy) allele to further increase AC [203,204] or inhibiting the expression of enzymes involved in amylopectin biosynthesis to increase the AC content [172,205,206]. In addition to the studies related to wheat starch properties, many researchers are providing new and efficient techniques that can improve the quality of bread wheat using molecular marker technology. Starch and protein have a major impact on the quality of flour products. Optimal starch and protein and the right levels of essential ingredients (iron, zinc, calcium, phosphorus, and antioxidants) are required in order to produce a healthy wheat product. Wheat starch metabolism should be studied in the anthesis and maturity stage. Looking at the climate changes in Korea or around the world, the stresses that affect starch metabolism are mostly high temperature and drought. Several pieces of research, such as on relative enzymes and metabolism, have been carried out regarding these stresses. When temperatures are elevated between anthesis to grain maturity, grain yield is reduced due to the reduced interval to capture resources. Among the resources, starch is especially sensitive to heat and drought stress compared to storage proteins. Both heat and drought stresses, which have a great influence on the grain filling period and anthesis, have a negative effect on starch synthesis. To improve wheat yield and quality under heat and drought stress, a thorough study of grain weight stability in terms of starch components and enzymes is needed. Sucrose and starch biosynthetic pathway mechanisms can alter the activity of an enzyme, and the changes to sucrose-metabolizing enzyme activities also modify the sucrose metabolism. Heat and drought stress suppress the starch deposition by lowering the activity of all enzymes such as sucrose phosphate synthase, sucrose synthase, ADP-glucose pyrophosphorylase, glucokinase, soluble starch synthase, and starch branching enzyme, which are involved in starch synthesis. Therefore, it is important to elucidate the mechanism of wheat starch synthesis in response to drought and high-temperature stress during the grain filling period. In recent years, many studies have revealed that most of these quality traits are undergoing development through genetic modification. The new information collected from hybrid and transgenic plants is expected to help develop novel starch for understanding wheat starch biosynthesis and commercial use. In addition, traditional breeding and genetic modification can be used together to produce new starches with modified properties. However, chemical or physical radiation-induced mutations can be accompanied by un-desirable and uncharacterized mutations in the whole genome [207,208]. Furthermore, RNAi-mediated interference of gene expression is often incomplete and transgene expression varies in different lineages. In addition, transgenic lines are considered genetically modified and must undergo a costly and time-consuming regulatory process [209]. Currently, wheat transformation research using plant genetic engineering technology is the main purpose of continuously controlling and analyzing the properties of wheat starch.

## Figures and Tables

**Table 1:** Classification of types of resistant starch (RS), food sources, and factors affecting their resistance to digestion in the colon [42,43].

Type of RS	Description	Example	Reference
RS1	Physically inaccessible starch	Whole or partly milled grains and seeds, legumes	[44]
RS2	Ungelatinized resistant granules with B-type crystallinity and are hydrolyzed slowly by α-amylases	Raw potatoes, green bananas, some legumes, high amylose starches	[44]
RS3	Retrograded starch (e.g., non-granular starch-derived materials)	Cooked and cooled potatoes, bread, cornflakes, food products with prolonged and/or repeated moist heat treatment	[45]
RS4	Chemically modified starches due to cross-bonding with chemical reagents, ethers, esters, etc.	Some fiber drinks, foods in which modified starches have been used (e.g., certain breads and cakes)	[46]
RS5	Amylose–lipid complex	Stearic acid-complexed high-amylose starch	[47]

**Table 2:** List of all functional markers available in wheat along with their KASP counterpart and standard cultivars for allele identification.

Trait	Gene	Marker	Allele	KASP ^a^	Standard	Reference
Gluten elasticity	*Glu-A1*	UMN19	*Glu-A1*(Ax1, Ax2 ^a^, AxNull)	gluA1.1_1594; gluA1.1_1883	Chinese Spring (CS), Opata 85	[129]
	*Glu-A1*	Ax2 ^a^	*Glu-A1b*(Ax2 ^a^)	As above	Pavon 76, Opata 85	[130]
	*Glu-B1*	TaBAC1215C06-F517/R964	*Glu-B1al*(Bx7^OE^)	Bx7^OE^	Dorico, ProINTA Colibr1, Klein Jabal	[131]
	*Glu-B1*	cauBx642	*Glu-B1b*(7 + 8); *Glu-B1i*(17 + 18); *Glu-B1h*(14 + 15)	NA	CS, Jing771, Pm97034	[132]
	*Glu-B1*	ZSBy9F2/R2	*Glu-B1f*(13 + 16)	NA	Baxter	[133]
	*Glu-B1*	ZSBy8F5/By8R5	*Glu-B1*(By8)	NA	Sunco	[133]
	*Glu-D1*	UMN25F/25R	*Glu-D1*(Dx2, Dx5)	Glu-D1d_SNP	CS, Pavon 76	[129]
	*Glu-D1*	UMN26F/26R	*Glu-D1*(Dy10, Dy12)	Glu-D1d_SNP	CS, Pavon 76	[129]
	*Glu-A3*	LA1F/SA1R	*Glu-A3a*	NA	Neixiang 188, Chinese Spring	[128]
	*Glu-A3*	LA3F/SA2R	*Glu-A3b*	NA	Gabo, Pavon 76	[128]
	*Glu-A3*	LA1F/SA3R	*Glu-A3c*	NA	Pitic, Seri 82	[128]
	*Glu-A3*	LA3F/SA4R	*Glu-A3d*	NA	Nidera Baguette 10, Cappelle-Desprez	[128]
	*Glu-A3*	LA1F/SA5R	*Glu-A3e*	NA	Amadina, Marquis	[128]
	*Glu-A3*	LA1F/SA6R	*Glu-A3f*	NA	Kitanokaori, Renan	[128]
	*Glu-A3*	LA1F/SA7R	*Glu-Ag*	NA	Bluesky, Glenlea	[128]
	*Glu-B3*	SB1F/SB1R	*Glu-B3a*	NA	Chinese Spring	[134]
	*Glu-B3*	SB2F/SB2R	*Glu-B3b*	NA	Renan, Gabo	[134]
	*Glu-B3*	SB3F/SB4R	*Glu-B3c*	NA	Insignia, Halberd	[134]
	*Glu-B3*	SB4F/SB4R	*Glu-B3d*	NA	Pepital, Ernest	[134]
	*Glu-B3*	SB5F/SB5R	*Glu-B3e*	NA	Cheyenne	[134]
	*Glu-B3*	SB6F/SB6R	*Glu-B3fg*	NA	Fengmai 27	[134]
	*Glu-B3*	SB7F/SB7R	*Glu-B3g*	NA	Splendor, Cappelle-Desprez	[134]
	*Glu-B3*	SB8F/SB8R	*Glu-B3h*	NA	Aca 303, Pavon 76	[134]
	*Glu-B3*	SB9F/SB9R	*Glu-B3ad*	NA	Opata 85	[134], Ikeda unpublished
	*Glu-B3*	SB10F/SB10R	*Glu-B3bef*	NA	Gawain	[134]
**Grain texture**	*Pina-D1*	Pina-N2	*Pina-D1a,b*	Pina-D1_INS	Chinese Spring, Zhongyou 9507	[135]
	*Pinb-D1*	Pinb-D1	*Pinb-D1a,b*	Pinb-D1_INS	Chinese Spring, Lorvin10	[136]
	*Pinb-D1*	Pinb-DF/Pinb-DR	*Pinb-D1p*	No	Shannongyoumai 3	[137]
	*Pinb-B2*	Pinb-B2v2	*Pinb-B2a, b*	Pinb2_IND	Chinese Spring, Zhongmai 175	[138]
**Amylose content**	*Wx-A1*	AFC/AR2	Null, Wild-type	NA	Norin 61, Kanton 107	[139]
	*Wx-B1*	BDFL/BRD	Null, Wild-type	WxB1_SNP	Norin 61, Kanton 107	[139]
	*Wx-D1*	BDFL/DRSL	Null, Wild-type	NA	Norin 61, California	[139]
**Wheat bread-making quality**	Wbm	NWPFor/Rev		Wbm_SNP	Mantol, Aca 601, Insignia	[140]

^a^ KASP (Kompetitive Allele-Specific PCR) markers are partially reported in Rasheed et al. [141]. Source: This table was modified by referring to He et al. [142]. OE indicates overexpressed.

**Table 3:** Recent status of wheat biotechnology research using characteristics of wheat starch.

Species	Target Gene	Target Trait	Results	Mutation System	Reference
Bread wheat	SBEII	Starch branching enzyme	Increased amylose/resistant starch contents	RNAi	[172]
Bread wheat	GBSSI, BMY, SSIII, SBEI, SBEIII, ISA3	Waxy protein (GBSSI), starch degrading (BMY), starch synthase (SSIII), starch branching enzyme (SBEI, SBEIII), isoamylase (ISA3)	Amylose/resistant starch variation	EMS	[173]
Bread wheat	TaWaxy	Granule-bound starch synthase	- Developed the induction of haploids/improved starch quality	SpCas9, lbCpf1, xCas9	[174]
Bread wheat	LMW-N13	Low-molecular-weight glutenin subunit (LMW-GS)	Superior dough properties (overexpression)	Agro-mediated transformation	[175]
Bread wheat	NAC019-A1	NAC transcription factor	Decreased starch granules	Agro-mediated transformation	[176]
Bread wheat	SBEIIa	Starch branching enzyme	Increased amylose/resistant starch contents	Cas9	[177]
Bread wheat	SPA-B	Storage protein activator (member of the bZIP family)	Decreased starch/glutenin content (overexpression)	Agro-mediated transformation	[178]
Bread wheat	Pinb-D1x	Puroindoline	Increased the kernel hardness and changed the internal structure of the kernel, flour properties variation (overexpression)	Bombardment	[179]
Bread wheat	bZIP28	Novel basic leucine zipper family	Decreased starch content	Cas9	[180]
Durum/ bread wheat	SBEIIa	Starch branching enzyme	Increased amylose/resistant starch contents	EMS	[181]
Durum wheat	SGP-1	Starch synthase	Increased amylose contents	EMS	[182]
Durum wheat	ATI	α-Amylase/Trypsin inhibitor	Reduced amount of potential allergens	Cas9	[183]
Durum wheat	SBEIIa	Starch branching enzyme	Increased amylose/resistant starch contents	RNAi	[184]
High-gluten spring wheat	Pinb, waxy, Agp2, SSIIa	Puroindoline (Pinb), waxy, AGPase (Agp2), starch synthase (SSIIa)	Obtained 1 novel allelic variation in the mutant lines-kernel hardness gene Pinb Frame shift and missense mutation of waxy and SSIIa-A: deleterious effects on their functions	EMS	[185]

## Data Availability

Not applicable.
